# Exploration of the path of digital technology empowering the sustainable development of alumni football—a study based on ordinal logistic regression analysis

**DOI:** 10.3389/fspor.2025.1613339

**Published:** 2025-08-20

**Authors:** Jiao Wang

**Affiliations:** ^1^ Faculty of Artificial Intelligence in Education, Central China Normal University, Wuhan, Hubei, China; ^2^ School of Physical Education, Hanjiang Normal University, Shiyan, Hubei, China

**Keywords:** digital technology, alumni football, sustainable development, path exploration, ordinal logistic regression

## Abstract

**Introduction:**

In the digital era, professional sports have rapidly embraced technologies such as big data, AI, and the Internet of Things to optimize performance, strategy, and fan engagement. However, the digital transformation of grassroots and amateur level sports remains significantly underdeveloped, posing a major obstacle to the inclusive and sustainable growth of national sports ecosystems. Alumni football, participated in by a vast and growing population of college graduates in China, emerges as a strategic gateway to bridging this digital divide.

**Methods:**

This study explores how digital technologies can empower the sustainable development of alumni football from the perspectives of data acquisition, processing, and application, with a focus on seven practical digital implementation scenarios. Using a questionnaire survey of 100 university football alumni and ordinal logistic regression analysis, ten digital factors were examined for their influence on alumni football development.

**Results:**

The results show that factors such as digital business models and digital team culture significantly contribute to sustainable development, whereas elements like virtual coaching and match data management have relatively limited impact.

**Discussion:**

This study not only addresses an urgent gap in digital grassroots sports integration but also provides replicable insights for policy makers, educators, and industry stakeholders aiming to promote large scale participation, cultural cohesion, and digital inclusion across broader segments of the sports domain.

## 1 Introduction

With the rapid development of internet technology, big data, cloud computing and other digital technologies have flourished, accelerating data collection, storage, analysis and application at an unprecedented rate. This has pushed China into a new stage of digital development (1, 2). The rapid advancement of digital technology is profoundly transforming the concepts, rules, systems, and methods of national governance, offering unprecedented opportunities for the development of various industries, including industry (3), agriculture (4), tourism (5), etc. Chinese sports culture, which reflects China’s rich traditional culture in the field of sports, encompasses a wide range of cultural forms related to sports. It not only bears the weight of history but also exhibits remarkable adaptability and resilience in response to the changing times. Entering the digital age, Chinese sports culture has been imbued with new strategic significance, becoming a vital vehicle for demonstrating national soft power and enhancing national cohesion (6, 7).

As the world’s foremost sport, football reflects a country’s comprehensive sports strength and has been integrated into national sports reorganization (8). Chinese football culture boasts a long history, with Cuju—football’s precursor—originating in China (9). Yet, in the era of modern football, its development has encountered numerous challenges. Compared to many footballing powers, Chinese football exhibits significant gaps in terms of competitive level, cultural popularity, and social influence (10, 11). When focusing on the leading football nations in Asia, it becomes apparent that China has fallen short in promoting and popularizing grassroots football, lacking the creation of a robust football culture. As a result, football has struggled to become a widely popular sport in China (12, 13).

**Figure 1:**
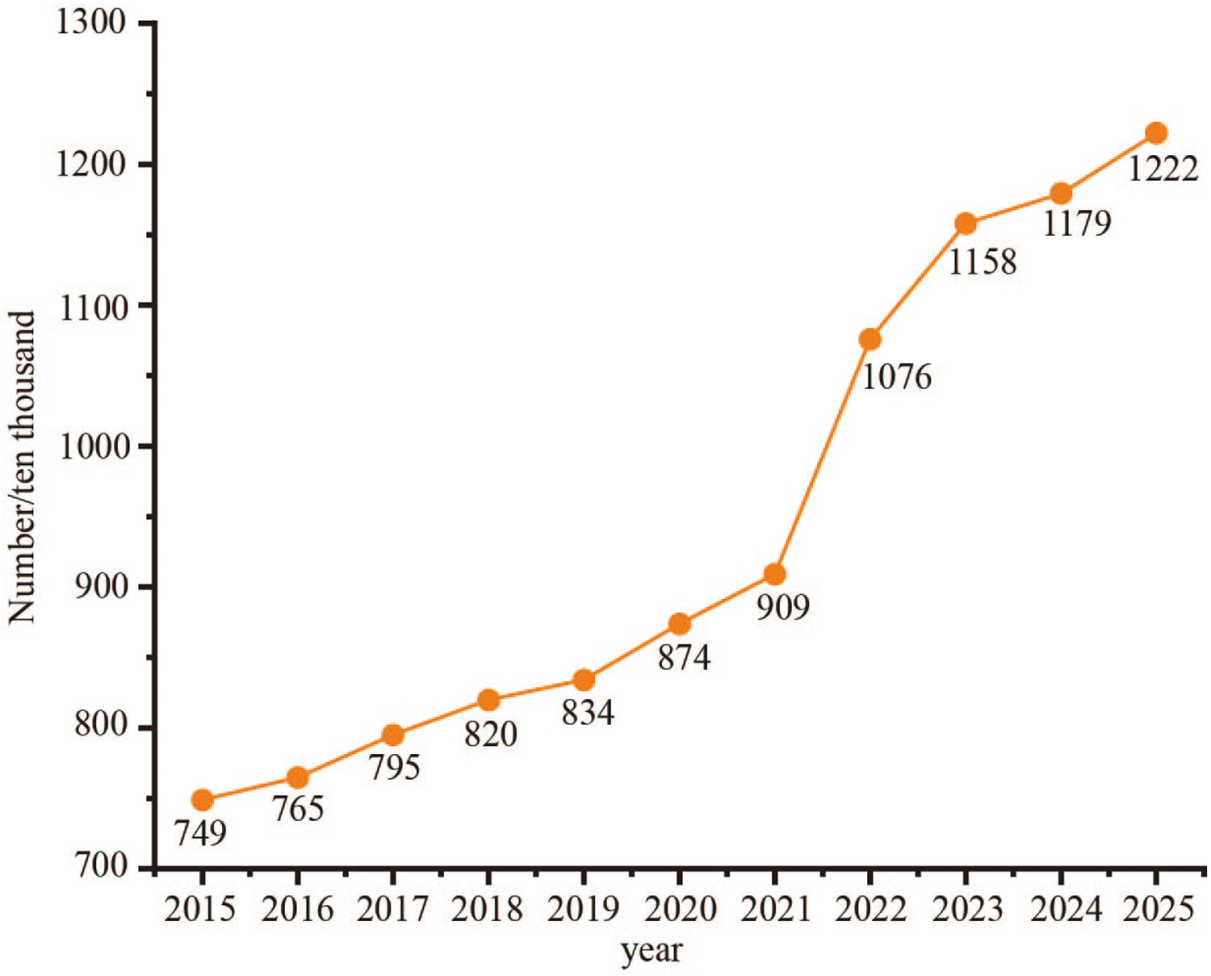
Number of college graduates in China from 2015 to 2025.

College graduates represent a key demographic driving the development of grassroots football. According to statistics released by China’s Ministry of Education, the number of college graduates is expected to reach 12.22 million by 2025, marking an increase of 5 million over the past decade, as shown in Figure 1. Every year, millions of college graduates enter society (14), many of whom possess a strong passion for football, thereby forming a vast alumni network. This network not only reflects the deep emotional connection alumni have with football but also plays a crucial role in promoting the growth and development of grassroots football. Given the rise of digital technologies, it is of great significance to tap into the immense potential of alumni football resources and enhance the study and practice of alumni football to elevate the overall level of Chinese football culture and promote the sustainable development of the sport.

In the context of digital transformation, professional football have widely adopted digital technologies to optimize their development. However, non-professional sports such as alumni football still lack sufficient digital empowerment. Alumni football serves as a crucial link connecting university graduates and grassroots sports, and it faces challenges such as an imperfect communication platform, a lack of professionalism, and lagging operation and promotion. Nevertheless, existing research rarely explores how digital technologies can address these issues to promote the sustainable development of alumni football.

Against the backdrop of existing studies predominantly focusing on digital technology applications in professional or youth sports, this research offers a novel perspective by centering on alumni football—a socially significant yet largely overlooked domain within grassroots sports. Firstly, by targeting the distinctive community of alumni football, it transcends the limitations of prior research that has primarily concentrated on professional football digitalization, marking the first systematic exploration of how digital technologies can enable the sustainable development of alumni football. Secondly, through ordinal logistic regression analysis, it precisely identifies core influencing factors such as digital business models and team culture digitalization from ten candidate digital factors, while clarifying that virtual coaching, event data statistics, and other elements are non-critical, thus providing a quantitative foundation for targeted interventions. Thirdly, integrating the Theory of Planned Behavior, it bridges digital technology application scenarios with the social attributes and emotional needs inherent in alumni football, proposing digital development strategies that balance practical utility and emotional connection. This work thereby fills a research gap in digital empowerment within non-professional sports contexts.

## 2 Literature review

### 2.1 Digital technology

Digital technology, accompanied by the iterative development of the internet, has given rise to a technical system designed to meet the diverse needs of the market. This system spans multiple stages, including data collection, transmission, processing, analysis, and application. It incorporates real-time data acquisition technologies, such as the Internet of Things (IoT) and smart sensor technologies, secure and efficient data transmission methods, 5G technology and internet communication protocols, as well as advanced data processing techniques, including big data and cloud computing technologies. Furthermore, it encompasses sophisticated data analysis tools, such as artificial intelligence (AI) and deep learning, alongside comprehensive data applications, including blockchain technology, digital twin technology, and interactive technologies. The interactions among these digital technologies are also cross-functional, as illustrated below:

(1) IoT Technology: Through intelligent perception, identification technologies, and pervasive computing, IoT combines various information-sensing devices with the internet, forming a vast network for information exchange and communication. This deep integration of the physical and digital worlds allows for real-time, accurate, and comprehensive data acquisition. Shao and He (15) utilized IoT technology to achieve real-time data collection and processing in his research, demonstrating that the model proposed can classify different types of faults in situations where parameter identification is difficult, thereby enhancing the accuracy and robustness of error detection in football matches. Based on IoT technology, Lu et al. (16) designed a motion tracking system capable of monitoring players’ foot motion and evaluating their skills while significantly reducing computing resource demands. Additionally, IoT technology has seen widespread application across various fields, including smart homes (17) and smart agriculture (18), where it has significantly improved management efficiency and service quality. Marinova-Kostova and Kostov (19) asserts that IoT technology plays a critical role in the digital transformation of enterprises, enabling interconnection between people and machines, a key component of Industry 4.0.
(2) 5G Technology: As the latest generation of cellular mobile communication technology, 5G features ultra-high speed, an enormous number of device connections, and ultra-low latency. This technology facilitates high-speed data transmission and supports large-scale device interconnections, with millisecond end-to-end latency. Dong (20), leveraging 5G communication technology in combination with virtual reality (VR), developed an intelligent sports teaching platform capable of guiding and correcting students’ body movements through 3D decomposition and playback. Moreover, 5G technology has demonstrated significant potential in fields such as telemedicine (21), autonomous driving (22), and virtual reality (23), contributing to the rapid expansion of the digital economy.
(3) Big Data Technology: Big data encompasses a range of technologies and methods for collecting, storing, managing, analyzing, and mining vast amounts of data. Its characteristics include high-speed processing, vast data volumes, and diverse data types. This technology enables the detection of complex correlations and latent values between data that may not be intuitively apparent, providing a scientific basis for decision-making in various domains. Fang et al. (24) argued that a successful football team is not solely composed of the players on the field but also includes the complete training, analysis, and coaching teams. The use of big data can predict offensive and defensive tactics for each position, thereby supporting football technology, tactical command, and decision-making. Additionally, big data technology is extensively employed in fields such as financial risk management (25), marketing (26), and clinical decision-making (27). Wong and Hinnant (28) highlighted that big data technology is progressively reshaping public policy frameworks and continues to disrupt various industries.
(4) Cloud Computing Technology: Cloud computing is an internet-based computing paradigm that provides dynamic, scalable, and often virtualized resources and services across networks, including servers, storage, databases, software development platforms, and networking services. Characterized by elastic scalability, resource pooling, on-demand services, and service billing, cloud computing significantly reduces IT costs for enterprises (29, 30). Li and Hong (31) discussed that traditional machine learning methods often upload football match results to centralized cloud servers, leading to issues such as network congestion and server delays. By uploading data from edge nodes to cloud servers and making fusion decisions, these problems can be alleviated. Additionally, cloud computing, when integrated with big data and virtual technologies, opens up new teaching opportunities in fields such as music (32) and dance education (33).
(5) AI Technology: AI simulates and extends human intelligence, including aspects such as learning, reasoning, self-correction, adaptability, knowledge representation, and natural language processing. AI is characterized by its strong data processing capabilities, independent learning abilities, and innovative problem-solving skills. McLean et al. (34) suggested that AI, as a tool for performance monitoring, fan and media engagement, and injury prediction, is poised to significantly impact the sports industry. Kulkarni (35) noted that AI’s focus in football matches surpasses even that of avid fans, as referees can track even the slightest movements of the ball and players through VAR technology. Additionally, AI has demonstrated substantial capabilities in assistive technologies for people with disabilities (36), and in extracting and utilizing complex medical data for outcome prediction, diagnosis, and treatment (37).
(6) Blockchain Technology: Blockchain is a distributed database that enables participants in a network to exchange and transmit secure, traceable, and immutable data without the need for a centralized authority. This technology ensures data security and reliability and has found widespread applications in the management and transaction of digital assets such as cryptocurrencies (38) and supply chain finance (39). Pu et al. (40) discussed the application of blockchain technology in collecting, storing, cleaning, mining, and visualizing the full cycle of football players’ injury data, combining it with machine learning algorithms to improve the monitoring system’s self-processing capabilities.
(7) Interactive Technology: Interactive technology focuses on enhancing the interaction between users and digital systems, including multimedia interaction, virtual reality (VR), and augmented reality (AR). Characterized by its intuitiveness, ease of use, naturalness, and personalization, interactive technology can be intelligently adjusted based on user behavior and preferences. Frederiksen et al. (41) developed a novel recreational soccer ball equipped with sensors to track its movement during fast-paced training games, encouraging users to engage in enjoyable physical exercise. Zhao and Guo (42) explored the application of VR technology in football training, noting how its integration with sports training overcomes limitations such as training ground availability and weather conditions, thus improving teaching and training methods.

### 2.2 Digital technology empowering the game of football

The development of modern football is no longer confined to the advancement of a singular athletic skill level but is increasingly characterized by the integration and empowerment of digital technologies, which evolve in tandem with the sport. The term “empowerment” refers to the strategic and intentional infusion of new capabilities into a given object to facilitate its functional optimization and the enhancement of its overall state. In this context, the empowerment process involves exploring and implementing novel methods or pathways to achieve specific objectives. In football, the application of digital technologies—such as sensing, interaction, and computer vision—not only provides valuable assistance in technical training for athletes, but also offers reliable tools for coaches to analyze players’ performance and enables referees to make accurate judgments during matches. Simultaneously, these technologies enhance the viewing experience for spectators. This paper reviews how digital technologies contribute to the development of football through seven practical scenarios commonly encountered in the sport.

#### 2.2.1 Player body monitoring and action prediction

In football, the use of digital technology to monitor and predict players’ physiological data in real-time offers coaches immediate, comprehensive feedback on player status. Additionally, it enables the analysis of player behavior patterns during games, providing robust data to support the formulation of game strategies. Rennie et al. (43) employed a GPS tracking and motion analysis system to monitor players’ movement, speed, and energy expenditure at various stages of the match. This analysis revealed how fatigue affects performance during different phases of the game, thus offering valuable insights for player rotation and training optimization. Seckin et al. (44) reviewed the use of wearable sensors in measuring and monitoring sports performance, injury prevention, rehabilitation, and overall performance enhancement, emphasizing that sensor technology can improve both training efficiency and match performance. Pokolm et al. (45) modeled a player’s scanning activity, quantifying head movements, gaze patterns, and spatial awareness using digital technology. The model provided insights into how players gather information prior to receiving the ball, as well as their subsequent actions, thus contributing to a better understanding of overall game performance. Aluneizi et al. (46) proposed a deep learning algorithm that combines RGB and pose estimation techniques to recognize football players’ movements, demonstrating that accurate and stable movement recognition can be achieved by integrating spatial and temporal flow representations.

#### 2.2.2 Tactical analysis and decision making

Coaches’ tactical analysis and decision-making processes are multifaceted, encompassing three primary dimensions: first, the formulation of tactics before the match based on the players’ condition and the opponent’s situation; second, the adjustment of tactics and strategies during the game, based on the analysis of players’ physical condition and on-field performance; and third, the dynamic optimization and adaptation of tactics, based on insights into the opposing team’s behavior under the coach’s strategic deployment. Nouraie et al. (47) explored a data-driven approach to identify the optimal team combination and individual player roles by analyzing extensive datasets, including performance indicators, game statistics, and opponent strategies. This method provides a foundation for building a competitive lineup. Fang et al. (24) also posits that the use of big data technology can predict the offensive and defensive tactics of individual players, thereby informing technical and tactical decision-making. AlMulla et al. (48) proposed a deep learning model named “SoccerNet,” based on a gated recurrent unit, which analyzes player performance at various stages of the game by processing information captured by the STATS platform within 15-min intervals. This data is then used to devise game strategies for both coaching staff and team management. Wang et al. (49) discussed the application of AI and machine learning to infer both the long-term and short-term intentions of opponents. This method enables dynamic tactical adaptation and strategic improvement based on analysis of player behavior and decision-making patterns, which was validated through its application to soccer games.

#### 2.2.3 Match reference assistance

In a formal football match, the officiating team consists of the referee, linesmen, fourth officials, and the video assistant referee (VAR). During the match, all decisions are supported by the collective input of these officials, with the final ruling made by the lead referee, which is considered final. Consequently, refereeing decisions have historically been a source of controversy in football. Celik (50) argued that the referees appointed by the governing committee could face competitive pressures, proposing a match-referee pairing model based on the Gale-Shapley algorithm, which was successfully applied in the Turkish professional football league. As the intensity of modern football increases, foul play has become more difficult to detect. To ensure fairness and reduce errors such as incorrect or missed judgments, digital technologies are increasingly being employed to assist referees. Hassan et al. (51) proposed an intention recognition system, utilizing computer vision and machine learning techniques to help referees determine whether a player intentionally handled the ball or made accidental contact. VAR technology (52, 53) provides referees with decision support via high-definition video replays and multi-angle footage analysis. Furthermore, Goal line technology (GLT) (54, 55), which uses sensors and image processing, can instantly and accurately verify whether the ball has crossed the goal line, effectively addressing the persistent issue of “ghost goals.” The semi-automatic offside technology (SAOT) (56), which integrates AI with refereeing, can automatically detect offside situations by tracking the movements and positions of players, further improving the fairness of the game. SAOT was implemented for the first time during the 2022 Qatar World Cup, using high-frequency AI technology to combine body and ball tracking data, enabling AI to determine offside positions (57).

#### 2.2.4 Score prediction system

Football has long been associated with the world of betting, where the ability to accurately predict match outcomes holds significant value for maximizing profits. The result of a football match is influenced by numerous complex factors, which often lack intuitive correlation with one another. However, each of these factors impacts the final outcome to varying degrees. Wheatcroft (58) utilized statistics from previous matches, such as shots on goal and corner kicks, as predictive data before the commencement of games. The Generalized Attacking Performance (GAP) and Bivariate Attacking (BA) models were proposed for this purpose, demonstrating their practical effectiveness. Building on data from the English Premier League’s 2022–2023 season, Loukas et al. (59) developed a regression algorithm based on big data technology, using historical team performance and Poisson distribution to predict goal numbers and match outcomes. In contrast to predictions derived from historical data, Klemp et al. (60) proposed an end-to-end system that utilizes raw and tracking data to forecast offensive and defensive movements, while also optimizing play decisions based on historical match data. Aarons et al. (61) employed real-time data and predictive analytics to forecast match outcomes in Australian football, leveraging live match data—such as player movement, team performance metrics, and contextual factors—along with machine learning algorithms to predict the likelihood of various outcomes in real-time.

#### 2.2.5 Optimize viewing experience

Football is a global sport with billions of fans, and the audience plays an indispensable role in the progression of the game. To enhance and deepen fans’ viewing experience, researchers have made significant investments in the development and upgrade of smart stadiums (62, 63). Beyond the construction of the physical stadium, Martins et al. (64) argued that emerging technologies and communication networks can increase fan interactivity during matches. This includes the integration of personal information, fan interaction systems, and detailed information about players, team tactics, and strategies, as well as interactive fan voting systems, all of which can serve to elevate fan engagement. Wang (65) proposed a deep learning-based key action recognition algorithm for football matches, enabling intelligent editing and highlight generation by automatically identifying critical events (such as goals, shots, corner kicks, and red or yellow cards). This system enhances the overall viewing experience for spectators.

#### 2.2.6 Scientific training program

Football is a physically demanding sport, requiring athletes to possess exceptional core physical abilities. The design of scientifically-based training programs has become essential, as it enables the systematic monitoring of data during training and the development of personalized training regimens. The application of intelligent sports equipment, powered by sensors, significantly enriches training methods. Yan (66) designed a flexible pressure sensor, using electrospun fiber materials, which adheres closely to the knee joint for gait monitoring during soccer training. Feng and Fan (67) embedded flexible sensors at eight specific locations inside soccer shoes, using voltage and pressure measurements to assess players’ shooting performance during training. Marshall et al. (68) employed VR technology to train players in heading skills, enabling them to develop the ability to head the ball without enduring the physical impact of repeated head collisions. Zhao and Guo (42) also explored the broad application of VR technology in soccer training. Regarding personalized training programs, Zhang and Ren (69) analyzed the challenges of personalized tracking and quantification within traditional teaching methods, proposing the development of digital teaching resources through big data analysis. This system would provide personalized resource recommendations to improve both the soccer skills and theoretical knowledge of students. Zafar et al. (70) applied a big data-driven approach to determine players’ running intensity and workload through personalized speed thresholds, ensuring that athletes’ training regimes meet the physical demands of the sport. Wang and Guo (71), Wang and Liu (72) utilized artificial intelligence technologies, including convolutional neural networks and machine learning, to recognize players’ movement characteristics, thereby offering personalized skill enhancement programs.

#### 2.2.7 Health management and injury prediction

The increasing frequency of matches, high-intensity running drills, and frequent acceleration and deceleration movements have collectively heightened the overall physical strain on players. Ensuring the health and well-being of athletes while minimizing injury risks has become a crucial area of research. Yang et al. (73) discussed the potential of big data technology in health management, suggesting its ability to monitor players’ exercise regimens in real-time by collecting data on exercise intensity, duration, blood oxygen levels, and blood pressure. Pillitter et al. (74) presented a real-world example of training decisions based on football training load data, elucidating the relationship between training load and injury risk. Pilka et al. (75) employed GPS-based wearable sensors to predict injuries by monitoring various metrics, such as players’ running distance, speed, acceleration, deceleration, and overall workload. These data are processed using machine learning algorithms to identify patterns linked to injury risks, enabling the implementation of preventive strategies to optimize training loads and reduce injury rates.

## 3 Current status

Empowered by digital technology, football is experiencing significant growth in the direction of datafication and diversification. Professional players benefit from advanced training equipment, data analysis, and intelligent management systems, which continually enhance their competitive abilities. Simultaneously, football talent development initiatives, such as youth training programs and professional campuses, are also flourishing due to the influence of digital technologies. However, not all players are able to transition into professional leagues, and for many, college football becomes the stage to continue pursuing their football dreams. With the influx of professional players, college football culture has gained unprecedented vitality, attracting an increasing number of students to participate. Upon graduation, these students, united by their passion for football and nostalgia for their campus experiences, form alumni teams that gather periodically for football activities, transcending time and space to connect based on school, region, or other factors. This phenomenon has given rise to alumni football, which not only fosters emotional bonds among alumni but also promotes exchange and collaboration among participants. Despite the rapid development of alumni football, several challenges remain.

### 3.1 Lack of alumni interaction platform and communication limitations

In the realm of alumni football, communication has traditionally relied on events organized by alumni associations across various regions, which have served as a communication bridge. However, the absence of dedicated platforms has led to several limitations. (1) The diverse personal backgrounds, career goals, and professional fields of alumni result in highly dispersed and uneven geographical employment patterns. This geographical dispersion hinders the ability of alumni to congregate and participate efficiently in local alumni football teams. (2) The fast pace of modern professional life makes it challenging for many alumni to commit to fixed training times, and there are often significant differences in football-related interests and schedules between teammates. Finding partners with compatible work and rest schedules, as well as similar football interests, presents a considerable challenge after leaving the local alumni football teams. (3) Local alumni football teams also face considerable limitations when it comes to cross-regional communication. While some universities invite local alumni teams back to campus to participate in events like the “Alumni Cup” as part of celebration activities, such opportunities are infrequent and spaced far apart. As a result, alumni often find it difficult to engage in cross-regional communication and collaboration. (4) Alumni football teams from different universities within the same region frequently miss opportunities to register for league and cup competitions due to the lack of effective communication platforms, thus missing chances for competition and interaction. (5) Data sharing across alumni football event platforms is often limited. The absence of a centralized, comprehensive platform to collect and integrate game data results in data islands. This issue hinders event planning, future planning, and the ability to share data with surrounding services such as hotels and catering, thereby limiting the potential for collaborative development within the associated Pindustrial chain.

### 3.2 Multi-dimensional lack of professionalism in alumni football

Alumni football, as a sport that combines physical exercise with social interaction, aims to improve individual physical fitness and football skills through participation while fostering alumni relations and promoting career cooperation. However, several issues persist concerning the professionalism of the sport. (1) Alumni participants are typically presumed to have some foundational football skills, but many lack structured training or warm-up routines. Players with lower skills often remain sidelined and miss opportunities to improve. This diminishes the sense of belonging and engagement among participants over time. (2) There is a lack of professional coaching, with tactical arrangements often based on the personal experiences of individual players rather than a structured understanding of teamwork and strategy. As a result, many players fail to grasp the importance of teamwork, which hampers the overall competitiveness of alumni football. (3) The equipment used to capture match footage is often inadequate. Most football stadiums are only equipped with single-angle surveillance cameras, which fail to capture the dynamic and high-energy moments of gameplay comprehensively. Even if multiple angles are available, extracting key frames is time-consuming, and the footage is not readily accessible for timely sharing on social platforms after the game. (4) Given that alumni typically participate in football activities during their leisure time, they are often physically fatigued, which increases the likelihood of strains or other injuries during intense running or physical confrontations. The lack of professional medical support or equipment at these events further exacerbates the risk of health-related issues. (5) Alumni football activities often include social aspects, such as post-game meals, where some participants neglect their physical recovery and consume excessive amounts of alcohol or cold drinks, which can lead to adverse health outcomes, including sudden cardiac events.

### 3.3 Lagging operation and promotion of alumni football

Alumni football teams are primarily organized on a voluntary basis, lacking clear management structures and organizational frameworks. This has resulted in a significant gap between the operational and promotional efforts of alumni football teams and those of professional clubs. (1) The funding for activities, such as matches and social gatherings, largely depends on voluntary contributions from individual alumni. This self-sustaining economic model limits the potential for expanding the scale of alumni football activities and hinders the long-term sustainability of these initiatives. (2) Publicity and promotion efforts for alumni football are often inadequate, with a lack of professional live broadcast capabilities and strategic publicity planning, which limits the sport’s visibility and brand influence. Even when live broadcasts are conducted, they tend to attract random viewers rather than targeted audiences. (3) There is a noticeable lack of innovation in content output, including event reports, player interviews, and fan interactions, which fails to fully engage the audience. This diminishes the likelihood of alumni football standing out on social media platforms or attracting significant attention from online traffic. (4) Alumni football activities are also lacking in terms of form innovation, with few diverse entertainment options available to appeal to the broader alumni community. The needs and experiences of potential participants, such as alumni families, are often overlooked. (5) Moreover, many alumni football teams have not established a strong team culture, including unified visual elements (such as team logos) or deep cultural meanings. The absence of a distinct cultural identity makes it difficult to foster a strong sense of pride and belonging among alumni, which also impedes the attraction of potential investors.

## 4 Methods

Based on the ways in which digital technology empowers football and the current status of alumni football, the index of “digital factors affecting the sustainable development of alumni football” has been refined. The Theory of Planned Behavior (TPB) (76) posits that behavioral intention is primarily influenced by three factors: attitude towards the behavior, subjective norms, and perceived behavioral control. This framework is particularly applicable to alumni football activities, as participation is voluntary and driven by personal motivation, social influence, and resource availability. Based on this theory, a total of 11 observation indicators have been selected, as shown in Table 1. With “whether digital technology can empower the sustainable development of alumni football” as the dependent variable and the 11 indicators, such as the digital alumni network platform, as independent variables, a measurement questionnaire was designed. Among them, the digital alumni network platform and the digital business model represent the perceived behavioral control, the digitalization of team culture and the social media content operation reflect the subjective norms, while the virtual reality experience and wearable devices reflect the attitude towards enhancing participation. Therefore, TPB provides a theoretical basis for variable construction and ensures the comprehensiveness and interpretability of the measurement model.

**Table 1:** Variable encoding and assignment.

Type	Variable name	Description	Encoding	Assignment
Dependent variable	Can digital technology empower the sustainable development of alumni football	An overall evaluation of whether digital technology can contribute to the sustainability of alumni football.	Y	Very disagree(1) → Very agree(5)
Independent variable	Age	Age groups of alumni.	$X_{1}$	20–29(1) → 60 and above(5)
	Digital alumni network platform	Integrating alumni information management, team daily operations, event registration and notifications, resource sharing and communication, etc., to provide a one - stop service for alumni football.	$X_{2}$	Very disagree(1) → Very agree(5)
	Intelligent wearable devices	Using intelligent wearable devices to collect physical data and provide personalized training plans and warm - up guidance for players through algorithm analysis.	$X_{3}$	Very disagree(1) → Very agree(5)
	Multi - angle video intelligent analysis	Deploying high definition cameras at multiple angles on football fields and extracting key frames and highlights from matches through video analysis technology.	$X_{4}$	Very disagree(1) → Very agree(5)
	Virtual coaches and tactical analysis	Based on digital resources, using AI technology to simulate professional coaches to provide tactical analysis, match strategy formulation, and other services for teams to enhance team competitiveness.	$X_{5}$	Very disagree(1) → Very agree(5)
	Health monitoring and warning system	Combining wearable devices and big data analysis technology to monitor players’ health in real - time and provide abnormal alarms and health management suggestions.	$X_{6}$	Very disagree(1) → Very agree(5)
	Event data statistics and management	Establishing a unified national alumni football event platform to collect and integrate match data for data analysis and mining.	$X_{7}$	Very disagree(1) → Very agree(5)
	Digital business model	Using blockchain technology to build diversified business models such as event crowdfunding, sponsorship management, and merchandise sales to provide stable funding and profit channels.	$X_{8}$	Very disagree(1) → Very agree(5)
	Social media content operation	Opening social media accounts to post player interviews, fan interactions, and other content to enhance team visibility and influence.	$X_{9}$	Very disagree(1) → Very agree(5)
	Virtual reality entertainment experience	Combining VR, AR, and other advanced technologies to create diverse entertainment forms, such as virtual viewing and interactive games, to enhance the participation experience and fun for alumni and their families.	$X_{10}$	Very disagree(1) → Very agree(5)
	Digitalization of team culture	Using AI technology to create a unique digital cultural system for teams, including the digital display and dissemination of elements such as logos, uniform designs, and slogans.	$X_{11}$	Very disagree(1) → Very agree(5)

In this questionnaire, there is no gender variable considering that most of the alumni football players are male. Considering that age differences would have an impact on the concept of exercise, the control variables of “20–29, 30–39, 40–49, 50–59, 60 and above” were set as “1, 2, 3, 4, 5”, respectively. For the main predictor variables that are more likely to affect the sustainable development of alumni football, such as “digital alumni network platform”, the values are also assigned in an increasing order of “1, 2, 3, 4, 5”, which respectively means “Very disagree, Disagree, General, Agree, Very agree”. The online questionnaire was distributed to the alumni football players through alumni networks for completion. A total of 100 participants were involved in the study. After screening, 88 valid responses were retained for further data analysis.

To ensure the representativeness and statistical validity of the sample, this study used the Slovin formula to determine the number of participants in the alumni football survey. The Slovin formula is applicable for calculating the sample size in a finite population. Its core logic is to determine the minimum sample size based on the error tolerance. The expression is as shown in Equation 1.

(1) $$n=\frac{N}{1+N\times e^{2}}$$

Where, n is the required sample size, N is the overall number of alumni football players, and e is the acceptable error range (set at 5% in this study).

A 2021 survey conducted by the Alumni Association of Tsinghua University showed that the average annual participation rate of offline alumni activities was approximately 0.3%, with sports-related activities accounting for less than one-third of that. Considering the physical demands and time costs of football, we assume that the actual number of alumni participating in football activities was 0.1%. Therefore, we can obtain the result as shown in Equation 2.

(2) $$N=1.222\times 10^{8}\times 0.1\%=12220$$

Substituting N=12,220 and e=0.05 into the Equation 1, we can obtain the required sample size as Equation 3.

(3) $$n=\frac{12220}{1+12,220\times {\left(0.05\right)}^{2}}\approx 387$$

Since the actual number of universities participating in alumni football activities across the country is far less than 0.1%, and due to limitations in terms of research time, budget, and the accessibility of the alumni network, the actual number of alumni that can be reached is limited. Therefore, we have adjusted the number of participants to 100, which is feasible.

All indices in the questionnaire were treated as ordinal variables with multiple categories ranging from low to high, and thus an ordinal logistic regression model was applied for statistical analysis. SPSS statistical software was employed for all data processing in this study, including reliability and validity tests, descriptive statistics, and regression analysis of the questionnaire results.

To ensure the reliability of the questionnaire, 30 alumni football participants were selected for a pre-survey in this study. The Cronbach’s alpha coefficient was used to test the internal consistency of the questionnaire (as shown in Equation 4). The results indicated that the overall Cronbach’s alpha coefficient of the questionnaire was 0.801, which was higher than the standard threshold of 0.7, indicating that the question items were well set and had good internal consistency, capable of reliably reflecting the characteristics of the measured variables. In terms of validity, three experts in sports management and digital technology were invited to evaluate the questionnaire items to ensure that each indicator accurately reflects the corresponding digital factors.

(4) $$\alpha =\frac{k}{k-1}\left(1-\frac{\sum \sigma_{y_{i}}^{2}}{\sigma_{y}^{2}}\right)$$

Where, k represents the number of questions in the questionnaire design, $\sum \sigma_{y_{i}}^{2}$ represents the total variance of all individual items, $\sigma_{y}^{2}$ represents the variance of the total score for all items. In this test, the three values are 50, 43, and 200.

## 5 Results

The age distribution characteristics of the respondents in this study were as follows: 20–29, 30–39, 40–49, 50–59, and 60 and above accounted for 36.4%, 26.1%, 28.4%, 5.7%, and 3.4% respectively (see Table 2). The data distribution of different age groups helps to reflect the different views of alumni of different ages on the recognition degree of digital technology. Therefore, the sample selection that can be used for this study is reasonable and representative. As can be seen from the data in the table, 76.1% of the views given by alumni are neutral or above. It can be seen that most alumni recognize that digital technology can have a positive impact on the sustainable development of alumni football.

**Table 2:** Digital technology empowers alumni football recognition basic description statistics table.

Type	Degree of recognition	Very disagree	Disagree	Neutral	Agree	Very agree	Classification summary	%
Age($X_{1}$)	20–29	1	4	9	11	7	32	36.4
	30–39	2	5	5	7	4	23	26.1
	40–49	1	5	10	7	2	25	28.4
	50–59	1	2	1	1	0	5	5.7
	60 and above	0	0	1	1	1	3	3.4
Recognition summary		5	16	26	27	14	88	100.0
%		5.7	18.2	29.5	30.7	15.9	100.0	

With “whether digital technology can enable the sustainable development of alumni football” as the dependent variable, “age ($X_{1}$)” as the control variable, and “$X_{2}-X_{11}$” as the main predictor, the ordinal logistic regression analysis was carried out. Model evaluation results were as follows: parallel line test $\chi^{2}=41.066$, significance probability = 0.512, greater than 0.05, so the model passed the comparative advantage hypothesis test. Meanwhile, the significance of -2 logarithmic likelihood value was p<0.001. Both Pearson and deviation significance probability p values were equal to 1, greater than 0.05. The pseudo $R^{2}$ values of Cox and Snell, Nagelkerke, and McFadden were 0.596, 0.629, and 0.306, respectively, which were normal and reasonable for cross-section data (see Table 3). Therefore, the ordinal logistic regression model constructed in this study passed the goodness of fit test.

**Table 3:** Model fit test table.

Index	Model fitting information		Degree of fitting		Pseudo $R^{2}$		
	Intercept only	−2 logarithmic likelihood value	Pearson	Deviation	Cox and Snell	Nagelkerke	McFadden
Standard value	260.831	181.032	213.833	181.032	0.596	0.629	0.306
Significance		< 0.001	1.000	1.000			

The regression results are shown in Table 4:

**Table 4:** Ordinal logistic model regression results.

Variable name		Parameter estimation	Standard deviation	Wald	Significance
$X_{2}$		0.466*	0.213	4.808	0.028
$X_{3}$		0.589**	0.227	6.743	0.009
$X_{4}$		0.574**	0.186	9.578	0.002
$X_{5}$		0.316	0.185	2.906	0.088
$X_{6}$		0.399*	0.201	3.92	0.048
$X_{7}$		0.374	0.207	3.256	0.071
$X_{8}$		0.866***	0.216	15.997	<0.001
$X_{9}$		0.143	0.193	0.549	0.459
$X_{10}$		0.644**	0.222	8.379	0.004
$X_{11}$		0.674**	0.215	9.803	0.002
$X_{1}$	“20–29” = 1	−1.615	1.235	1.71	0.191
	“30–39” = 2	−1.179	1.255	0.882	0.348
	“40–49” = 3	−1.781	1.313	1.84	0.175
	“50–59” = 4	−2.294	1.635	1.969	0.161
	“60 and above” = 5	0			

Note: *expression p<0.05, **expression p<0.01, ***expression p<0.001.

**Figure 2:**
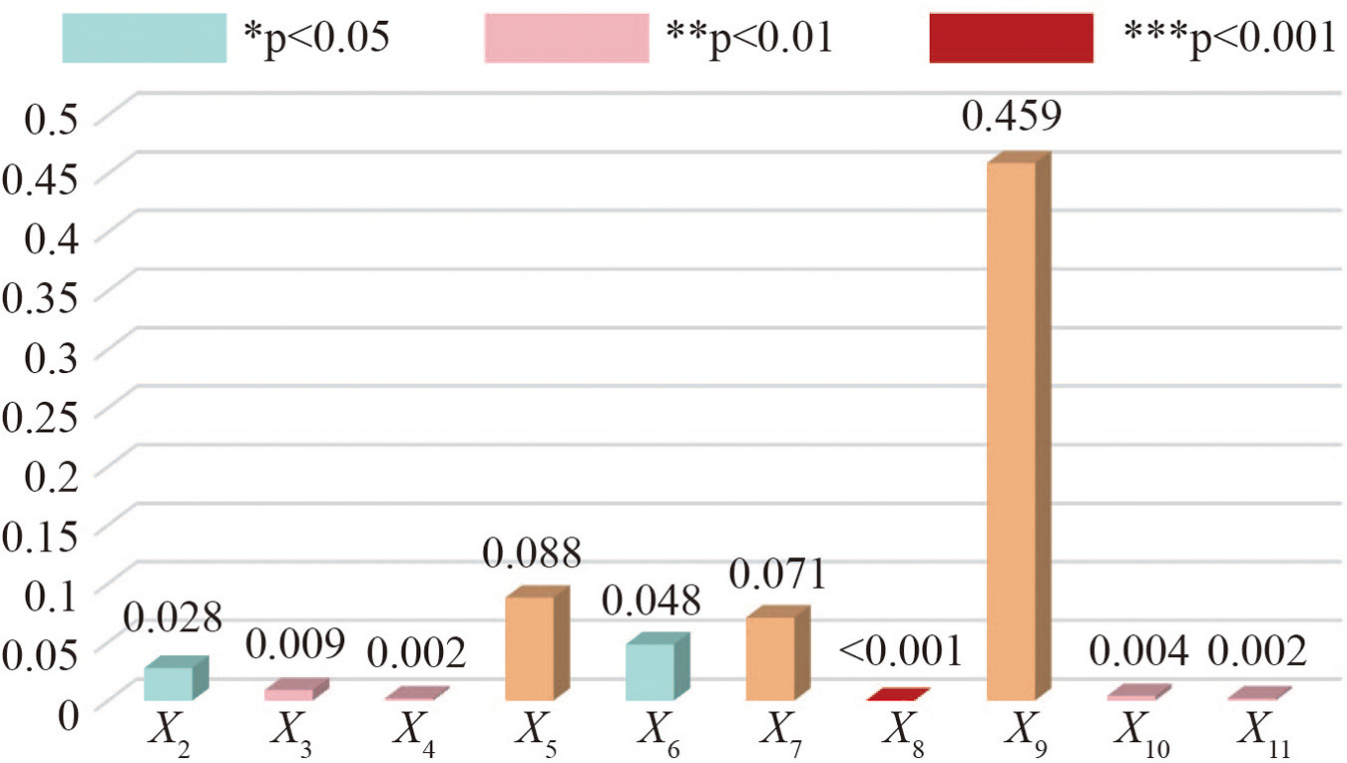
Regression coefficients and significance of the numerical factors.

As can be seen from the results of regression analysis in Table 4, the indicators of significance affecting the sustainable development of alumni football are $X_{8}$, $X_{11}$, $X_{4}$, $X_{10}$, $X_{3}$, $X_{2}$ and $X_{6}$ in descending order. Although there are differences among different ages in the recognition degree of whether digital technology can enable the sustainable development of alumni football, the significance coefficient is all greater than 0.05. Therefore, it can be considered that age is not the main factor affecting the development of alumni football. Additionally, from Figure 2, it can be intuitively seen that the three indicators $X_{5}$, $X_{7}$ and $X_{9}$ of the predictive variables have not significant influence on the sustainable development of alumni football, so they can be considered as not the main factors affecting the development of alumni football.

The regression results indicate that $X_{9}$ have no significant impact on the sustainable development of alumni football, a phenomenon rooted in the fact that alumni football primarily functions as a platform for emotional connection and physical exercise rather than a commercialized sports event. Most participants prioritize offline interactions and team cohesion over online exposure or fan engagement, resulting in low dependence on social media operations. Similarly, $X_{5}$ can be attributed to the amateur nature of alumni football: unlike professional teams that rely on sophisticated tactical systems, alumni teams place greater emphasis on recreational participation and social bonding than competitive performance, thus reducing the demand for virtual coaching tools. As for$X_{7}$, their lack of significance may stem from the fragmented and irregular nature of alumni football activities. In contrast to professional leagues with standardized data collection systems, alumni events are often temporary and decentralized, making unified data management less critical to their sustainable development.

## 6 Discussion

According to the theory of planned behavior, the main factors influencing behavioral intention include attitude, subjective norms, and perceived behavioral control. When an individual’s attitude is more positive, the support from others is stronger, and the perception of behavioral control is higher, the intention to act becomes stronger, and vice versa. Therefore, any factor that influences alumni’s perceptions of participation in alumni football, subjective norms, and perceived behavioral control can alter the degree to which alumni identify with the sport.

(1) The digital business model is critical for alumni football’s sustainable development, as it leverages alumni’s passion for football while delivering tangible benefits. Blockchain technology enables a transparent, decentralized fund management system, accurately recording and verifying sponsorships, ticket revenue, tournament winnings, and donations. This ensures transparency and fosters trust in fund usage. Additionally, an online crowdfunding platform can be established to raise funds for specific event needs—such as prize money, venue rentals, and transportation costs—by clearly outlining the goals and allocation of each fund and creating a reasonable return mechanism linked to capital investment. The collection of event data, audience behavior data, and fan preferences can be analyzed using big data technology, optimizing advertising and sponsorship strategies by matching advertisers with their target audiences. The innovative fund management and profit models mitigate the risks associated with reliance on a single income source, expanding revenue channels and ensuring the long-term viability of alumni football. From the participants’ perspective, alumni can financially support the sport they love while receiving direct benefits through reward mechanisms (such as souvenirs, VIP tickets, sponsor rights, etc.), which further strengthens their identification with the sport. Moreover, the digital business model can attract additional investors and sponsors by improving the transparency and operational efficiency of the competition, creating a virtuous cycle that supports the sustainable development of alumni football.
(2) The digitalization of team culture is also a significant factor in enabling the sustainable development of alumni football. As demonstrated by the “Red Devil spirit” of Manchester United and the “White Legend spirit” of Real Madrid, a team’s culture must be preserved to lay a foundation for long-term development. Digital technology can generate unique cultural symbols, such as logos, slogans, and other cultural elements, and integrate historical traditions, core values, and spiritual connotations into digital content. This content can then be disseminated via social media, online platforms, and virtual reality channels to broaden the team’s cultural influence. Additionally, by leveraging the cherished memories of alumni from their university days, digital projects such as virtual memorial halls, historical review videos, and interactive cultural displays can be constructed. These initiatives allow alumni to revisit their team’s glorious history and cultural heritage on digital platforms, strengthening their sense of belonging, identity, and emotional connection. Moreover, the digitalization of team culture can promote alumni interaction through online activities, virtual football celebrations, and e-sports events, which not only stimulate alumni participation but also foster deeper emotional resonance and further consolidate the team culture.
(3) Multi-angle video intelligent analysis also plays a significant role in the sustainable development of alumni football. Alumni football matches typically last around two hours, and intelligent video analysis can substantially reduce the costs associated with comprehensive event recording. By using multiple high-definition cameras and intelligent analysis systems, key moments in the game—such as goals, saves, and key passes—can be automatically identified and captured through key frame extraction technology. This ensures that these moments are clearly recorded and can be easily replayed and analyzed. Furthermore, video analysis can be used for game replays, allowing alumni to review footage from different angles to assess individual performance and tactical execution. Motion trajectory analysis technology can track players’ running routes, speed variations, and positional distribution, providing valuable data to inform subsequent training adjustments. It also helps extract highlights for easy sharing on social media.
(4) The virtual reality entertainment experience is another important digital factor driving the sustainability of alumni football. Alumni participation in football is not only influenced by personal initiative but also by family support, which plays a critical role. Through VR/AR technology, a cross-generational football social space can be created, fostering family collaborative competition. For example, interactive experiences such as gesture-controlled virtual penalty shootouts with children or spouses participating in tactical discussions via AR glasses can enhance emotional connections within alumni families and their engagement with football. Additionally, VR technology enables a “cloud home” viewing experience that transcends time and space limitations. Alumni can enjoy a panoramic 270${}^{\circ }$ view via 8K ultra-high-definition quality and 3D spatial audio technology, capturing real-time tactical positioning details of players. The ambient vibration feedback device enhances realism by recreating the tactile sensations of the arena, further increasing immersion. AR technology can complement the viewing experience by overlaying real-time data, such as player statistics and game metrics, enhancing interaction and engagement. The virtual reality entertainment experience not only diversifies game viewing formats but also provides additional opportunities for alumni and their families to participate, thereby supporting the sustainable development of alumni football.
(5) Smart wearables are also critical digital factors promoting the sustainability of alumni football. Devices such as IoT-enabled jerseys, smartwatches, and smart insoles have become integral tools in football. These devices can monitor athletes’ physiological data in real-time, including heart rate, stride frequency, distance covered, and calories burned. This provides players with accurate feedback to help optimize their training programs and reduce the risk of overtraining and sports injuries. For alumni football, smart wearables enhance participants’ awareness and management of their health status, offering personalized, data-driven paths for health improvement, thereby encouraging long-term participation and the sustainable development of the sport. By continuously monitoring and providing feedback, players can adjust their training intensity and recovery strategies according to their physical condition, improving overall athletic performance and fitness. Additionally, these devices enable real-time data upload to the cloud, and through integration with social platforms, alumni can share their athletic results, motivating each other, strengthening the spirit of competition, and fostering team cohesion.

## 7 Conclusion and suggestion

### 7.1 Research conclusion

(1) Unlike the use of digital technology to improve athletes’ physical and tactical skill, the empowerment of digital technology in alumni football places greater emphasis on building digital business models, shaping the unique cultural atmosphere of the team, and enhancing the overall technological ecosystem experience. For alumni, the application of digital technology is primarily aimed at achieving economic and other benefits, which aligns more closely with their current reality than with the demands of high-level competition.
(2) The majority of alumni exhibit a positive attitude toward the role of digital technology in promoting the sustainability of alumni football. According to the survey results, 76.1% of respondents expressed a neutral or above approval attitude, indicating that the vast majority of alumni recognize the potential of digital technology in this field at a cognitive level.
(3) The regression results reveal that digital factors such as the digital business model, the digitalization of team culture, multi-angle video intelligent analysis, virtual reality entertainment experiences, smart wearable devices, digital alumni network platforms, and health detection and early warning systems have significant positive effects on the sustainable development of alumni football. In contrast, digital factors such as virtual coaching, tactical analysis, game statistics management, and social media content operations are not central to the sustainability of alumni football.

### 7.2 Countermeasure and suggestion

Based on the regression results, the following suggestions are made for the sustainable development of alumni football, enabled by digital technology, from three levels: government, enterprise, and university.

(1) Government level: strengthening policy guidance and infrastructure support. The government should focus on enhancing policy support and infrastructure for alumni football. Through top-level design, the government should establish and improve the policy framework for integrating digital technology with sports activities. This includes providing financial support, policy guidance, and platform resources to enterprises and universities. A special fund should be set up to encourage and fund the application of technologies such as smart wearable devices, sports data analysis, and video analysis in alumni football event organization, player training, and health monitoring. It is also important to include alumni data platforms in the “new infrastructure” plan and establish data security grading standards to ensure compliance with sensitive data handling. Moreover, fostering close collaboration between alumni football organizations, local governments, enterprises, and universities is essential. Cross-departmental cooperation should be promoted, integrating social resources to facilitate the development of digital sports technologies. Initiatives such as digital sports technology exhibitions and industry forums should be organized to promote technological collaboration and innovation. Additionally, innovative events, such as virtual reality football leagues and blockchain-based score systems, should be held to create a digital ecosystem for venue booking, event broadcasting, and health management through the “Urban Sports Brain” platform, thus enabling greater alumni participation.
(2) Enterprise level: building technological ecosystem and business model innovation. Enterprises should recognize the importance of digital technology in alumni football and actively participate in its digital transformation. They should work towards creating a comprehensive “hardware + software + service” solution. Attention should be given to hardware development, with joint efforts to create high-performance digital products, such as wearable devices equipped with myoelectric sensors. Software development should focus on breakthroughs in technologies like 3D motion capture algorithms and deep learning analysis. Furthermore, service offerings should be strengthened to provide advanced technical solutions that improve event management efficiency and the competitive level of alumni football. Enterprises should also collaborate with alumni football organizations to develop digital business models, such as online platforms and virtual arenas, creating new revenue streams for alumni football. This would promote the diversification and commercialization of football content, expand new business areas, and enhance market influence. Through digital platforms, enterprises can provide advertising and sponsorship opportunities, strengthen the brand’s market presence, and contribute to the social responsibility of alumni football.
(3) University level: deepening the integration of education, production, and emotional bonds. Universities, as key centers of education and research, should actively build multidimensional cooperation with the government and enterprises to explore innovative applications of digital technology in alumni football. On one hand, universities should promote the research and development of digital technologies, and on the other, they should explore how these technologies can meet the practical needs of alumni football. As the spiritual home of alumni, universities must take on the responsibility of providing long-term support and interaction platforms for alumni. Establishing a digital alumni network platform is crucial, offering continuous communication channels for alumni, updating platform content, and providing services such as game information and technology sharing. This will ensure that more alumni can easily participate in various alumni football activities. Regular events such as alumni football games, technical exchanges, and symposia should be organized to strengthen alumni connections and enhance their sense of belonging. Additionally, universities should organize large-scale events like the Alumni Football Festival to engage alumni, faculty, students, and external stakeholders, further promoting the popularization and development of alumni football. To fully leverage alumni involvement, universities can establish an alumni mentor system, inviting outstanding alumni to participate in digital technology research and development projects, thereby supporting the innovation and practical advancement of alumni football technology.

## Data Availability

The original contributions presented in the study are included in the article/Supplementary Material, further inquiries can be directed to the corresponding author/s.
